# UACR adjustment attenuates associations of selected routine blood markers with documented diabetic retinopathy more than eGFR adjustment: a cross-sectional study in type 2 diabetes

**DOI:** 10.3389/fendo.2026.1983060

**Published:** 2026-10-27

**Authors:** Jing Zhang, Mingliang Xu, Baoyi Xiong, Baohua Luo, Long Pang

**Affiliations:** 1 The Second Clinical College of Guangzhou University of Chinese Medicine, Guangzhou, Guangdong, China; 2 Department of Ophthalmology, Guangdong Provincial Hospital of Chinese Medicine, The Second Affiliated Hospital of Guangzhou University of Chinese Medicine, Guangzhou, Guangdong, China

**Keywords:** covariate adjustment, diabetic retinopathy, estimated glomerular filtration rate, FIB-4 index, serum albumin, type 2 diabetes, urinary albumin-to-creatinine ratio

## Abstract

**Background:**

Studies of routine blood markers and diabetic retinopathy often adjust for estimated glomerular filtration rate (eGFR), although albuminuria captures a different aspect of kidney damage. We compared these associations under eGFR and urinary albumin-to-creatinine ratio (UACR) adjustment.

**Methods:**

We analyzed 8,159 adults hospitalized with type 2 diabetes from January 2020 to March 2026. Primary exposures were serum albumin, total bilirubin, fibrinogen and the FIB-4 index. Four models were fitted in a fixed complete-case sample, with additional adjustment for eGFR, log UACR, or both. The outcome was documented diabetic retinopathy (DR), based on ophthalmological assessment and discharge diagnosis; DR = 0 included unassessed patients without a DR diagnosis. Prevalence ratios were estimated by marginal standardization.

**Results:**

Among 3,978 complete-case patients, 958 (24.1%) had documented DR. Adjustment for log UACR attenuated the serum albumin association more than adjustment for eGFR (73.3% versus 34.3%; difference 38.9 percentage points, 95% CI 30.7 to 49.0) and moved the fibrinogen estimate close to one. Binary albuminuria produced substantially less attenuation than continuous log UACR. Multiple imputation supported the renal-adjustment comparison, although the albumin interval included the null after mutual renal adjustment. Serum albumin remained associated with documented DR after restriction to eGFR ≥90 mL/min/1.73 m^2^ (prevalence ratio 0.762, 95% CI 0.649 to 0.873), whereas no clear evidence of an eGFR association was observed within this stratum (0.981, P = 0.823). Conclusions: In this single-center inpatient cohort, eGFR and UACR adjustment yielded different estimates for the studied blood-marker associations with documented DR. Attenuation denotes proportional reduction in the absolute log prevalence-ratio estimate, not a causal proportion explained. Dichotomizing UACR discarded information and reduced attenuation in these models.

## Introduction

Diabetic retinopathy is a common microvascular complication of type 2 diabetes and remains a major cause of visual impairment. A global meta-analysis estimated its prevalence among people with diabetes at 22.3% (1). Longer diabetes duration and poorer glycemic control are consistently associated with greater retinopathy risk (2, 3). Alongside these established factors, routinely measured blood markers are increasingly examined as correlates of retinopathy because they are inexpensive and readily available in clinical datasets.

The four blood markers provide contrasting biological contexts for this comparison. Serum albumin has reported inverse associations with retinopathy and reflects nutritional and inflammatory influences (4–6). Fibrinogen provides a positive acute-phase and coagulation-related counterpart to albumin (7). Total bilirubin has an inverse observational association with retinopathy and experimentally demonstrated antioxidant activity (8, 9). FIB-4 combines age, aminotransferases and platelet count into a score developed for liver fibrosis assessment and used in diabetes care (10, 11). Comparing these markers allows us to examine whether sensitivity to renal adjustment is shared across different routine blood measurements; none is specific to a single biological pathway.

Renal status is particularly relevant. eGFR describes filtration function, whereas urinary albumin-to-creatinine ratio (UACR) captures albuminuric kidney damage; current chronic kidney disease classification incorporates both dimensions (12, 13). eGFR alone may not capture the same renal information as UACR when blood markers are modeled in relation to retinopathy.

Previous studies have compared the renal measures as exposures, finding stronger associations of albuminuria than eGFR with vascular complications, retinopathy severity and retinal ischemia (14, 15). The remaining question is how choosing eGFR, UACR or both as adjustment variables changes the estimated associations between routine blood markers and retinopathy.

We compared associations of serum albumin, total bilirubin, fibrinogen and FIB-4 with documented diabetic retinopathy under eGFR adjustment, UACR adjustment and mutual adjustment in a hospital-based cohort of adults with type 2 diabetes. Secondary comparisons examined continuous and dichotomized renal measures and restriction by eGFR. FIB-4 component and renal-phenotype analyses were exploratory.

## Materials and methods

### Study design and participants

This single-center retrospective cross-sectional study included adults with type 2 diabetes admitted to the Department of Endocrinology of Guangdong Provincial Hospital of Chinese Medicine between January 2020 and March 2026 (n = 8,159). Type 2 diabetes was based on the clinical diagnosis recorded at admission. For patients with multiple admissions, only the first eligible admission was retained. The study size was determined by the available eligible records within this period; no *a priori* sample-size or power calculation was performed. Reporting follows the STROBE statement for cross-sectional studies (16).

The study was approved by the Ethics Committee of Guangdong Provincial Hospital of Chinese Medicine (ZE2026-335-01), which waived the requirement for informed consent because the analysis used pre-existing, de-identified clinical data.

### Outcome

The outcome was documented diabetic retinopathy (DR). Ophthalmology consultation for fundus examination was routinely requested for patients hospitalized with diabetes in the endocrinology department, but completion could not be verified for every participant. DR = 1 required an ophthalmological assessment identifying DR and a DR discharge diagnosis. Patients without a DR discharge diagnosis were coded DR = 0, including those who had not completed ophthalmological assessment. Thus, DR = 0 denotes no documented DR, not confirmed absence of retinal disease. Individual examination status and severity grading were unavailable in the analysis dataset.

### Exposures and additional comparisons

Blood measurements were taken from the first available laboratory results after admission, usually from fasting samples collected the following morning. The primary exposures were serum albumin, total bilirubin, fibrinogen and the FIB-4 index, calculated as age × aspartate aminotransferase/(platelet count × √(alanine aminotransferase) (10). Age was retained as a covariate in the FIB-4 models because it is also strongly associated with diabetic retinopathy. The FIB-4 estimates in these models therefore represent associations conditional on age rather than the performance of the unadjusted clinical score. Globulin, calculated as total protein minus albumin, was examined as an additional plasma-protein marker.

Fibrinogen was examined alongside albumin because both are synthesized by hepatocytes but respond differently during the acute-phase response. The individual components of FIB-4 were examined in exploratory analyses to describe their associations with documented DR.

### Renal indices

eGFR was abstracted directly from hospital laboratory reports. The laboratory information system used the 2009 CKD-EPI creatinine equation (17) throughout the study period; the study team did not generate the primary eGFR values. These reported values were retained to represent renal function as recorded in the source data. UACR used the first morning urine sample obtained after admission. The hospital laboratory measured urinary albumin by immunonephelometry using Siemens reagents on a BN II analyzer (Siemens), and urinary creatinine by an enzymatic method using Roche reagents on a Modular PPI analyzer (Roche). The two measurements were performed on separate platforms in the same laboratory, and values were abstracted from laboratory reports. Recorded units were μmol/L for serum creatinine, mg/L for urinary albumin and μmol/L for urinary creatinine; UACR was expressed in mg/g. Laboratory values were used to characterize renal status, without using clinical nephropathy or chronic kidney disease labels as covariates.

### Covariates

Models were adjusted for age, sex, diabetes duration, glycated hemoglobin, systolic blood pressure and body mass index. These variables represent demographic background, diabetes chronicity, glycemic status, blood pressure and body size, providing a common adjustment set for comparing renal specifications. They do not establish a sufficient causal adjustment set; contemporaneous metabolic measures may also reflect disease or treatment.

The models used laboratory renal measures rather than the discharge diagnosis of diabetic nephropathy. That diagnosis was strongly associated with documented DR, but this alone does not establish how it was assigned (**Supplementary Table S7**). Smoking status was missing in 44.9% of records. Hypertension diagnosis was not included in addition to systolic blood pressure. Recorded long-term preadmission treatment and cardiovascular-history variables were not included in the primary models; restriction to statin non-users was examined in a sensitivity analysis (**Supplementary Table S2**).

### Statistical analysis

The main analysis compared the four blood-marker associations across a common base model, eGFR adjustment, log UACR adjustment and mutual renal adjustment. A fixed complete-case sample with observed exposures, renal indices and covariates allowed comparison without changes in sample composition.

Because documented DR was common, associations are reported as prevalence ratios rather than odds ratios (18). For each marker contrast, logistic marginal standardization compared the average predicted probability after increasing each participant’s marker value by one fixed complete-case standard deviation with the average predicted probability at that participant’s observed marker value, leaving covariates unchanged. Individual PR intervals exponentiated the 2.5th and 97.5th percentiles of log PR from 1,000 non-parametric bootstrap row resamples. Two-sided Wald P values used log PR divided by its bootstrap standard error and the standard normal distribution. Robust-variance Poisson estimates (19) are diagnostic results in **Supplementary Table S10**.

Total bilirubin, fibrinogen, FIB-4 and UACR were natural-log transformed because of right-skewed distributions. Marker effects are expressed per 1-standard-deviation increase; eGFR is expressed per 10 mL/min/1.73 m^2^.

Four models were fitted. Model 1 adjusted for age, sex, diabetes duration, glycated hemoglobin, systolic blood pressure and body mass index. Model 2 added eGFR, model 3 added log UACR, and model 4 included both renal measures. Predictor-design collinearity in the FIB-4 models was assessed using variance inflation factors (VIFs) from auxiliary regressions of each predictor on the remaining predictors with an intercept (**Supplementary Table S3B**).

Model-3/model-2 PR ratios used 2,000 shared bootstrap resamples, with percentile intervals computed on the log-ratio scale and exponentiated. Attenuation was the proportional reduction in the absolute log PR estimate relative to model 1, expressed as 100 × [1 − |log PR(Mk)|/|log PR(M1)|]. Its intervals and between-model differences used percentiles on the percentage-point scale from 1,000 shared resamples. These complete-case model contrasts are reported with intervals, without hypothesis-test P values. They do not estimate causal mediation or a proportion of disease or association explained; instability is addressed in **Supplementary Table S11**.

Secondary analyses compared four representations of renal status, binary albuminuria (UACR ≥30 mg/g), a binary composite of UACR ≥30 mg/g or eGFR <60 mL/min/1.73 m^2^, continuous eGFR and continuous log UACR. As a further secondary comparison of adjustment with restriction, analyses were repeated in nested groups with eGFR ≥60 and ≥90 mL/min/1.73 m^2^. Marker effects in these strata were standardized using the standard deviation from the full analytic sample.

Baseline continuous variables are reported as mean ± standard deviation or median (25th to 75th percentile), as appropriate, and were compared with Welch’s t test or the Mann–Whitney U test. Categorical variables are reported as n (%) and were compared with the Pearson chi-squared test. P values are two-sided and unadjusted for multiplicity, and 95% intervals are pointwise. Secondary and exploratory findings are interpreted descriptively rather than as confirmatory tests. Unadjusted association estimates are provided in **Supplementary Table S9**.

### Sensitivity and exploratory analyses

Globulin, individual FIB-4 components, renal phenotypes defined by eGFR and UACR thresholds, and duration-by-UACR prevalence patterns were examined as exploratory analyses. Sensitivity analyses assessed alternative samples, adjustment variables, estimation methods, missing-data handling and outcome perturbation.

Sensitivity analyses for serum albumin excluded hypoalbuminemia, restricted the sample to eGFR ≥60 mL/min/1.73 m^2^, and added hemoglobin to the model; hemoglobin was treated as a sensitivity covariate because anemia may reflect nephropathy, inflammation and nutritional status. Other checks included exclusion of markedly abnormal transaminases, stratification by sex, restriction to statin non-users and exclusion of macroalbuminuria (**Supplementary Table S2**). A *post hoc* acute-phase analysis added white cell count (WCC) and the neutrophil-to-lymphocyte ratio (NLR) to M1 and M3; C-reactive protein and procalcitonin were unavailable in the analysis dataset (**Supplementary Table S8**). A forward perturbation analysis applied hypothetical false-negative and false-positive outcome changes, including sensitivity varying by albuminuria status, to the observed DR coding. This assessed changes in the serum albumin model-3 estimate, not correction of actual ascertainment bias (**Supplementary Table S13**). During revision, a sensitivity analysis replaced reported eGFR with the 2021 race-free CKD-EPI creatinine estimate (20) in models 2 and 4 for the four primary markers, holding the original 3,978 participants, exposure standard deviations and other model specifications fixed (**Supplementary Table S14**).

### Missing data

The primary analysis retained a common complete-case population with observed exposures, renal indices and covariates for comparisons across all four models. This design does not remove selection bias. UACR was available for 5,804 of 8,159 participants (71.1%). Multiple imputation by chained equations was used as a sensitivity analysis (21), restricted to 7,420 participants with observed DR coding, serum albumin, age and sex; 739 lacked albumin, while the other entry variables were complete. Within this population, 2,110 participants required UACR imputation. The imputation model included the outcome, all five blood markers, both renal measures and the base covariates; total bilirubin, fibrinogen, FIB-4 and UACR were natural-log transformed. After 20 burn-in iterations, 10 datasets were retained at 10-iteration intervals. During revision, the analysis was extended from model 3 to all four models. Model log prevalence ratios and paired model-3-minus-model-2 contrasts were pooled using Rubin’s rules with t-based inference and within-imputation variances from 1,000 shared bootstrap resamples. Full specifications and included-versus-excluded comparisons are reported in **Supplementary Tables S1** and **S5**.

Prothrombin time and gamma-glutamyl transferase were examined initially but were not included in the primary analysis because of measurement reliability and data-completeness concerns, respectively (**Supplementary Table S6**).

### Software

Analyses used Python 3.12, statsmodels 0.14.6, scipy 1.17.1, pandas 3.0.2 and numpy 2.4.4. Figures were produced with matplotlib. OpenAI Codex (desktop application, GPT-5; OpenAI) assisted with coding and statistical-reporting checks; the authors reviewed the code and verified the results. Analysis code, package versions, seeds and run instructions are available from the corresponding author upon reasonable request. Rerunning the analyses requires authorized access to the study data.

## Results

### Participants

The cohort included 8,159 hospitalized patients with type 2 diabetes, of whom 1,619 (19.8%) had documented diabetic retinopathy. The fixed complete-case sample contained 3,978 patients, including 958 (24.1%) with documented DR, compared with 661 of 4,181 excluded participants (15.8%; **Figure 1**). UACR was the most frequently missing key variable; other sources of missingness are summarized in **Figure 1** and **Supplementary Material**. In the selection model adjusted for age, sex, systolic blood pressure, eGFR and diabetes duration, documented DR was associated with UACR availability (OR 1.357, 95% CI 1.164–1.582; P < 0.001; n = 7,019; **Supplementary Table S5**). The eGFR ≥60 and ≥90 mL/min/1.73 m^2^ strata included 3,111 and 1,817 patients, respectively.

**Figure 1:**
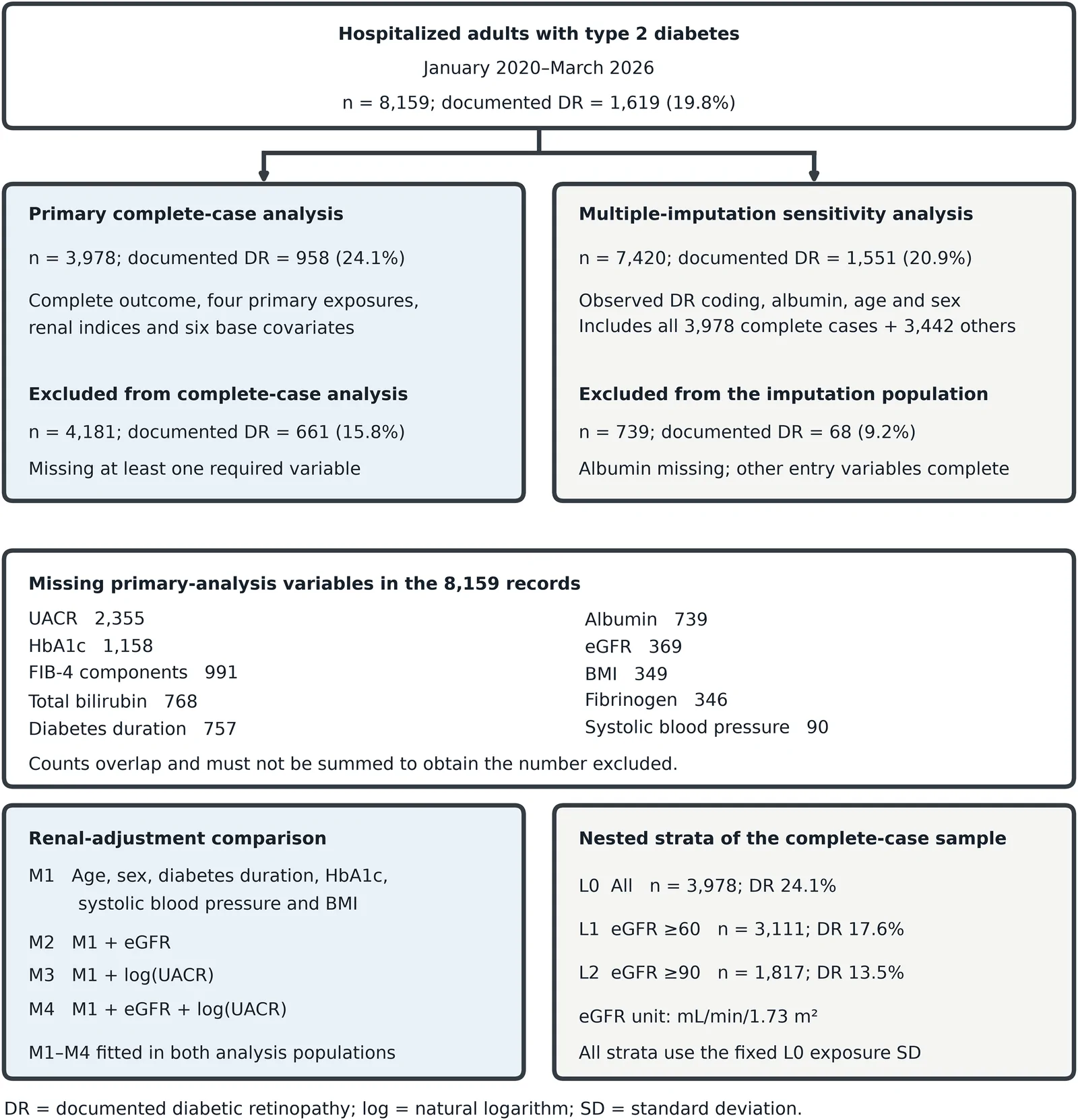
Participant flow and analytic structure. The study dataset comprised 8,159 adults hospitalized with type 2 diabetes. Complete-case analysis included 3,978 patients; 4,181 were excluded because at least one required variable was missing. Multiple-imputation analysis included 7,420 patients with observed DR coding, serum albumin, age and sex, comprising all 3,978 complete cases and 3,442 additional patients. The remaining 739 patients lacked albumin. Variable-specific missing counts overlap and cannot be summed. The eGFR strata were nested within the complete-case sample and used hospital-reported eGFR. All four models were fitted in both the complete-case and imputation analyses. DR, documented diabetic retinopathy; eGFR, estimated glomerular filtration rate; UACR, urinary albumin-to-creatinine ratio; BMI, body mass index; HbA1c, glycated hemoglobin.

Patients with documented DR were older, had longer diabetes duration and higher systolic blood pressure, and had lower eGFR than those without documented DR (**Table 1**). Median UACR was 120.59 (25.00–883.32) mg/g in patients with documented DR and 12.64 (5.01–36.86) mg/g in those without documented DR (P < 0.001). Glycated hemoglobin was slightly lower in the documented DR group (8.69% versus 8.85%, P = 0.043). Serum albumin and total bilirubin were lower, whereas fibrinogen and FIB-4 were higher, in patients with documented DR.

**Table 1:** Baseline characteristics of the fixed analytic sample (n = 3978).

Variable	All (n = 3978)	Documented DR (n = 958)	No documented DR (n = 3020)	Test statistic	P
Age, years	62.76 ± 12.98	67.06 ± 11.69	61.39 ± 13.08	t = 12.71, df = 1779	< 0.001
Male, n (%)	2168 (54.5)	491 (51.3)	1677 (55.5)	χ^2^ = 5.37, df = 1	0.021
Diabetes duration, years	10.19 ± 7.97	15.95 ± 7.73	8.37 ± 7.14	t = 26.95, df = 1511	< 0.001
Systolic blood pressure, mmHg	135.34 ± 20.06	139.78 ± 21.63	133.93 ± 19.32	t = 7.48, df = 1473	< 0.001
Body mass index, kg/m^2^	24.20 ± 3.84	23.99 ± 3.65	24.26 ± 3.89	t = −1.97, df = 1703	0.049
Glycated hemoglobin, %	8.81 ± 2.30	8.69 ± 2.12	8.85 ± 2.35	t = −2.03, df = 1761	0.043
Hypertension, n (%)	2207 (55.5)	714 (74.5)	1493 (49.4)	χ^2^ = 185.41, df = 1	< 0.001
Statin use, n (%)	775 (19.5)	246 (25.7)	529 (17.5)	χ^2^ = 30.89, df = 1	< 0.001
Insulin use, n (%)	1876 (47.2)	715 (74.6)	1161 (38.4)	χ^2^ = 382.27, df = 1	< 0.001
Renal indices					
eGFR, mL/min/1.73 m^2^	81.62 ± 26.77	64.99 ± 29.87	86.90 ± 23.36	t = −20.77, df = 1348	< 0.001
UACR, mg/g	18.31 (6.24–79.86)	120.59 (25.00–883.32)	12.64 (5.01–36.86)	U = 2267574, Z = 26.51	< 0.001
Primary exposures					
Serum albumin, g/L	40.75 ± 4.23	38.68 ± 4.72	41.40 ± 3.84	t = −16.21, df = 1381	< 0.001
Total bilirubin, µmol/L	9.70 (6.90–12.90)	8.40 (5.50–11.67)	10.10 (7.40–13.30)	U = 1098568, Z = −11.24	< 0.001
Fibrinogen, g/L	3.11 (2.66–3.72)	3.46 (2.86–4.17)	3.03 (2.61–3.59)	U = 1812662, Z = 11.82	< 0.001
FIB-4 index	1.18 (0.86–1.65)	1.34 (0.98–1.84)	1.13 (0.82–1.58)	U = 1731929, Z = 9.21	< 0.001
Additional variables					
Globulin, g/L	27.73 ± 4.65	28.16 ± 4.85	27.60 ± 4.57	t = 3.17, df = 1534	0.002
Hemoglobin, g/L	132.77 ± 19.33	122.93 ± 21.60	135.90 ± 17.44	t = −16.92, df = 1375	< 0.001

Values are mean ± standard deviation, median (25th to 75th percentile) or n (%). Continuous variables were compared using Welch’s t test or the Mann-Whitney U test, as appropriate; categorical variables were compared using Pearson chi-squared tests. P values are descriptive and were not corrected for multiple comparisons. DR, documented diabetic retinopathy; eGFR, estimated glomerular filtration rate; UACR, urinary albumin-to-creatinine ratio. DR = 0 indicates no documented DR and includes patients without completed ophthalmological assessment.

### Secondary analysis by eGFR restriction

After adjustment for the base covariates, all four primary markers were associated with documented DR (**Table 2**). The serum albumin prevalence ratio was 0.694 (95% CI 0.655–0.731) in the full analytic sample, 0.753 (0.688–0.824) at eGFR ≥60 mL/min/1.73 m^2^ and 0.762 (0.649–0.873) at eGFR ≥90 mL/min/1.73 m^2^. The FIB-4 association also persisted across these strata. By contrast, the associations of total bilirubin and fibrinogen weakened and were no longer significant in the eGFR ≥90 stratum.

**Table 2:** Prevalence ratios after progressive restriction by estimated glomerular filtration rate.

Marker	L0, all (n = 3978)	L1, eGFR ≥ 60 (n = 3111)	L2, eGFR ≥ 90 (n = 1817)	L2 with eGFR additionally adjusted
Serum albumin	0.694 (0.655–0.731), P < 0.001	0.753 (0.688–0.824), P < 0.001	0.762 (0.649–0.873), P < 0.001	0.761 (0.651–0.873), P < 0.001
Total bilirubin	0.759 (0.714–0.802), P < 0.001	0.843 (0.775–0.915), P < 0.001	0.931 (0.826–1.051), P = 0.245	0.931 (0.825–1.049), P = 0.247
Fibrinogen	1.243 (1.186–1.297), P < 0.001	1.178 (1.096–1.268), P < 0.001	1.089 (0.966–1.225), P = 0.173	1.089 (0.966–1.225), P = 0.173
FIB-4 index	1.103 (1.039–1.165), P = 0.001	1.120 (1.029–1.205), P = 0.006	1.237 (1.111–1.368), P < 0.001	1.237 (1.109–1.366), P < 0.001
Globulin (non-albumin protein control)	1.065 (1.010–1.118), P = 0.016	1.024 (0.950–1.107), P = 0.543	1.014 (0.889–1.145), P = 0.819	1.013 (0.889–1.144), P = 0.832
eGFR, per +10 mL/min/1.73 m^2^	0.836 (0.820–0.854), P < 0.001	0.868 (0.820–0.923), P < 0.001	0.981 (0.828–1.159), P = 0.823	—
Documented DR prevalence	24.1%	17.6%	13.5%	—

Values are adjusted prevalence ratios (95% confidence intervals) from marginal standardization. Marker contrasts use one SD from L0 (n = 3978), held constant across strata and models; bilirubin, fibrinogen and FIB-4 use natural-log SDs. eGFR contrasts are per 10 mL/min/1.73 m^2^. L0, all complete cases; L1, eGFR ≥60; L2, eGFR ≥90 mL/min/1.73 m^2^. SD, standard deviation; eGFR, estimated glomerular filtration rate.

The association between eGFR and documented DR weakened with restriction: the prevalence ratio per 10 mL/min/1.73 m^2^ was 0.836 (0.820–0.854) in the full sample, 0.868 (0.820–0.923) at eGFR ≥60, and 0.981 (0.828–1.159; P = 0.823) at eGFR ≥90. Additional adjustment for eGFR within the ≥90 stratum had little effect on the albumin or FIB-4 estimates.

### Comparison of eGFR and UACR adjustment

In the same 3,978 patients, the serum albumin prevalence ratio changed from 0.694 (0.655–0.731) in model 1 to 0.787 (0.745–0.828) after eGFR adjustment and 0.907 (0.861–0.954) after log UACR adjustment (**Table 3**; **Figure 2**). Adding eGFR to the UACR-adjusted model produced little further change (model 4, 0.919).

**Table 3:** Prevalence ratios across four model specifications in the fixed analytic sample.

Marker	Model 1, base covariates	Model 2, + eGFR	Model 3, + log UACR	Model 4, + both
Serum albumin	0.694 (0.655–0.731), P < 0.001	0.787 (0.745–0.828), P < 0.001	0.907 (0.861–0.954), P < 0.001	0.919 (0.871–0.966), P = 0.002
Fibrinogen	1.243 (1.186–1.297), P < 0.001	1.113 (1.055–1.168), P < 0.001	0.994 (0.940–1.045), P = 0.812	0.980 (0.926–1.030), P = 0.437
Total bilirubin	0.759 (0.714–0.802), P < 0.001	0.856 (0.806–0.900), P < 0.001	0.915 (0.866–0.964), P < 0.001	0.930 (0.879–0.977), P = 0.005
FIB-4 index	1.103 (1.039–1.165), P = 0.001	1.090 (1.026–1.149), P = 0.004	1.084 (1.027–1.139), P = 0.003	1.082 (1.024–1.136), P = 0.004
Globulin (non-albumin protein control)	1.065 (1.010–1.118), P = 0.016	0.983 (0.932–1.033), P = 0.500	0.975 (0.931–1.019), P = 0.283	0.960 (0.916–1.006), P = 0.092

Values are prevalence ratios (95% confidence intervals) per one SD from L0 (n = 3978; documented DR = 958), held constant across all four models. Bilirubin, fibrinogen and FIB-4 use natural-log SDs. Estimates use marginal standardization. M1, base covariates; M2, +eGFR; M3, +log UACR; M4, +both. SD, standard deviation; eGFR, estimated glomerular filtration rate; UACR, urinary albumin-to-creatinine ratio.

**Figure 2:**
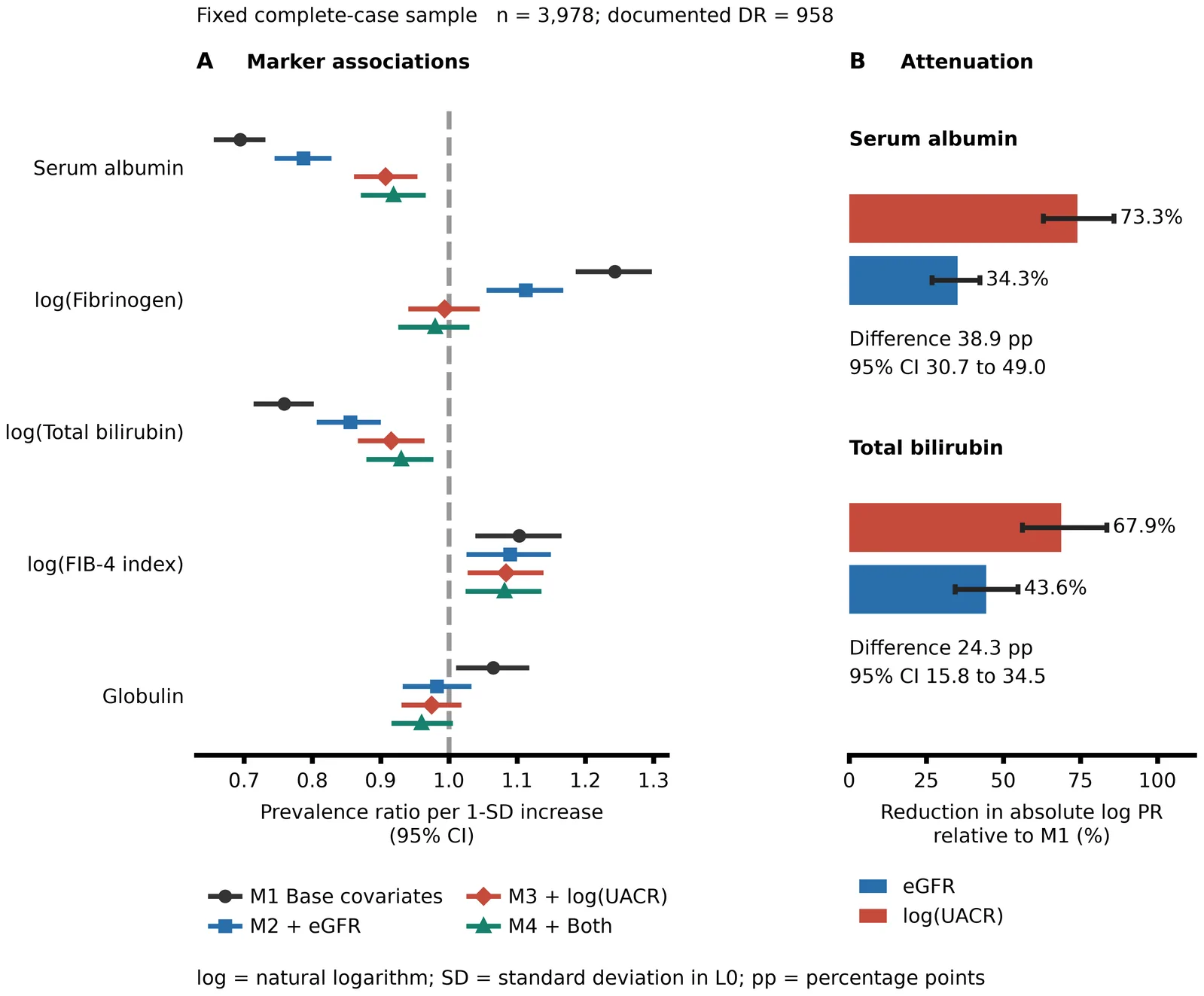
Comparison of eGFR and UACR adjustment. Both panels use the fixed analytic sample (n = 3,978; 958 with documented diabetic retinopathy). **(A)** Prevalence ratios for five markers under four models estimated by marginal standardization. Model 1 adjusted for age, sex, diabetes duration, glycated hemoglobin, systolic blood pressure and body mass index; model 2 added eGFR; model 3 added log UACR; and model 4 added both renal indices. Natural-log-transformed markers are labeled directly on the y-axis. Every marker contrast uses one standard deviation from the fixed complete-case sample. **(B)** Proportional reduction in the absolute log PR estimate relative to model 1 after adding eGFR or log UACR for serum albumin and total bilirubin. Error bars are 95% bootstrap percentile confidence intervals from 1,000 resamples. Paired UACR-minus-eGFR differences and their intervals are shown in percentage points. These contrasts are descriptive, without multiplicity adjustment. eGFR, estimated glomerular filtration rate; UACR, urinary albumin-to-creatinine ratio. DR, documented diabetic retinopathy.

Fibrinogen changed from 1.243 (1.186–1.297) in model 1 to 1.113 (1.055–1.168) after eGFR adjustment and 0.994 (0.940–1.045; P = 0.812) after UACR adjustment. Total bilirubin was attenuated but remained associated with documented DR after UACR adjustment (0.915 [0.866–0.964], P < 0.001). FIB-4 changed little across the four models.

For serum albumin, the proportional reduction in the absolute log PR estimate was 73.3% (95% CI 63.0–85.8) after log UACR adjustment and 34.3% (26.9–42.3) after eGFR adjustment; the difference was 38.9 percentage points (30.7–49.0) (**Table 4**). The corresponding difference for total bilirubin was 24.3 percentage points (15.8–34.5). Paired model-3/model-2 PR ratio intervals excluded one for serum albumin, fibrinogen and total bilirubin and included one for FIB-4.

**Table 4:** Comparison of eGFR and UACR adjustment.

Comparison	Point estimate	95% CI	P
Reduction in absolute log PR (%) and difference (percentage points)			
Serum albumin, by eGFR	34.3%	26.9%–42.3%	—
Serum albumin, by log UACR	73.3%	63.0%–85.8%	—
Serum albumin, difference (percentage points)	38.9	30.7–49.0	—
Total bilirubin, by eGFR	43.6%	34.3%–54.7%	—
Total bilirubin, by log UACR	67.9%	56.2%–83.5%	—
Total bilirubin, difference (percentage points)	24.3	15.8–34.5	—
Ratio of the model 3 estimate to the model 2 estimate			
Serum albumin	1.153	1.120–1.187	—
Fibrinogen	0.893	0.870–0.916	—
Total bilirubin	1.069	1.044–1.096	—
FIB-4 index	0.994	0.973–1.017	—
Renal index entered alone and after mutual adjustment			
eGFR entered alone, per +10 mL/min/1.73 m^2^	0.836	0.820–0.854	< 0.001
eGFR after adding log UACR	0.951	0.930–0.974	< 0.001
log UACR entered alone, per 1 SD	1.652	1.596–1.708	< 0.001
log UACR after adding eGFR	1.571	1.504–1.641	< 0.001

All estimates use the fixed analytic sample and base covariates. Attenuation is the proportional reduction in the absolute log PR estimate relative to model 1, expressed as a percentage, not a causal proportion explained. Each individual attenuation percentage and each between-adjustment difference is accompanied by its own 95% confidence interval. Percentage intervals are expressed in percent; difference intervals are in percentage points. These percentile intervals use 1,000 shared resamples; model-3/model-2 PR ratio intervals use 2,000 shared resamples on the log scale, then exponentiation. Dashes indicate that no hypothesis-test P value is reported for these paired contrasts. P values in the final section are two-sided normal Wald tests using bootstrap standard errors of individual log PRs. eGFR, estimated glomerular filtration rate; SD, standard deviation; UACR, urinary albumin-to-creatinine ratio.

Mutual adjustment showed the same asymmetry. Adding log UACR changed the eGFR prevalence ratio from 0.836 to 0.951, whereas adding eGFR changed the log UACR prevalence ratio from 1.652 to 1.571 (**Table 4**).

### Secondary comparison of renal-variable specification

Renal-variable specification materially affected the serum albumin estimate (**Table 5**; **Supplementary Table S12**). The proportional reduction in the absolute log PR estimate was 32.5% with binary albuminuria and 29.9% with the binary composite, compared with 34.3% for continuous eGFR and 73.3% for continuous log UACR.

**Table 5:** Effect of renal-variable specification on association attenuation.

Renal index added to model 1	Serum albumin PR (95% CI)	M1 log-PR attenuated
None (model 1)	0.694 (0.655–0.731)	0.0%
Binary albuminuria, UACR ≥ 30 mg/g	0.782 (0.741–0.821)	32.5%
Binary composite, UACR ≥ 30 mg/g or eGFR < 60	0.774 (0.734–0.814)	29.9%
eGFR, continuous	0.787 (0.745–0.828)	34.3%
log UACR, continuous	0.907 (0.861–0.954)	73.3%

Prevalence ratio per 1-standard-deviation increase in serum albumin, estimated by marginal standardization in the fixed analytic sample. The underlying renal measurements and analytic sample were identical across these specifications; only their representation in the model changed. eGFR, estimated glomerular filtration rate; PR, prevalence ratio; UACR, urinary albumin-to-creatinine ratio. Attenuation is the proportional reduction in the absolute log PR estimate relative to model 1, expressed as a percentage.

### Exploratory globulin comparison

Globulin was weakly associated with documented DR in model 1 [1.065 (1.010–1.118), P = 0.016] but not after adjustment for either eGFR or log UACR.

### Exploratory FIB-4 component analyses

The FIB-4 association was examined in exploratory component analyses (**Table 6**). When entered separately, aspartate aminotransferase and platelet count were not associated with documented DR, whereas alanine aminotransferase was inversely associated [0.876 (P < 0.001)]. When all three components were entered together, alanine aminotransferase remained inversely associated (0.747, P < 0.001), aspartate aminotransferase became positively associated (1.195, P < 0.001), and platelet count remained null.

**Table 6:** Associations of the three FIB-4 components with documented diabetic retinopathy.

Component	Entered alone	All three entered together
Aspartate aminotransferase (log)	0.983 (0.926–1.037), P = 0.543	1.195 (1.106–1.292), P < 0.001
Alanine aminotransferase (log)	0.876 (0.821–0.930), P < 0.001	0.747 (0.679–0.822), P < 0.001
Platelet count	0.982 (0.929–1.032), P = 0.506	0.974 (0.920–1.025), P = 0.348
FIB-4 index (composite, for reference)	1.103 (1.039–1.165), P = 0.001	—

Values are prevalence ratios (95% confidence interval) per 1-standard-deviation increase, estimated by marginal standardization and adjusted for the base covariates. FIB-4 components were examined separately and together in exploratory analyses of their associations with documented DR. Full models are given in **Supplementary Table S4**.

The inverse alanine aminotransferase association was consistent with its position in the denominator of FIB-4. These component models describe associations and do not quantify each component’s contribution to the composite association or establish a biological mechanism.

### Robustness analyses

Multiple-imputation analysis included 7,420 participants. UACR adjustment produced greater attenuation than eGFR adjustment for serum albumin and total bilirubin. The paired ratios of the model-3 to model-2 prevalence ratios were 1.155 (95% CI 1.127–1.183) and 1.063 (1.040–1.086), respectively (both P < 0.001), with both underlying estimates below one. The serum albumin estimate was 0.955 (0.915–0.997; P = 0.037) after UACR adjustment and 0.969 (0.929–1.010; P = 0.141) after mutual renal adjustment. Full four-model results, comparisons and diagnostic details are reported in **Supplementary Table S1**.

Serum albumin sensitivity analyses were directionally consistent. The UACR-adjusted association remained after excluding hypoalbuminemia and after restriction to eGFR ≥60 mL/min/1.73 m^2^; additional adjustment for hemoglobin weakened it to 0.949 (0.897–1.000; P = 0.064) (**Supplementary Table S2**). In the *post hoc* acute-phase analysis (n = 3,974), adding WCC and NLR moved the serum albumin PR from 0.695 to 0.719 in M1; with both inflammation proxies and UACR adjustment, the PR was 0.914 (95% CI 0.866–0.965; P = 0.002; **Supplementary Table S8**).

In the corrected forward perturbation analysis, the 11 scenario mean albumin PRs ranged from 0.909 to 0.946; 3 of 200 realizations reached or exceeded one in the scenario with sensitivity 0.70 and specificity 0.95. These simulations do not exclude real-world ascertainment bias (**Supplementary Table S13**).

Estimator diagnostics are reported in **Supplementary Table S10**. In the FIB-4 models, VIFs were approximately 1.55 for log(FIB-4) and 1.92 to 2.54 for age, with a maximum of 2.54 across all predictors (**Supplementary Table S3B**). Using the 2021 CKD-EPI equation, the UACR-versus-eGFR attenuation differences remained positive for serum albumin (38.2 percentage points, 95% CI 30.2–48.3) and total bilirubin (23.4, 15.0–33.2) in the fixed analytic sample (**Supplementary Table S14**).

### Exploratory renal-phenotype and prevalence analyses

Exploratory cross-classification of eGFR and UACR at standard KDIGO thresholds showed marked differences in documented DR prevalence (**Table 7**, **Figure 3A**). Prevalence was 33.3% among patients with eGFR ≥60 mL/min/1.73 m^2^ and UACR ≥30 mg/g (P2), compared with 20.6% among those with eGFR <60 and UACR <30 mg/g (P3). The adjusted prevalence ratio for P2 versus P3 was 1.579 (95% CI 1.230–2.147, P = 0.001). Patients with eGFR ≥60 and UACR <30 mg/g nevertheless accounted for 24.3% of all documented DR cases.

**Table 7:** Exploratory renal-phenotype associations with documented diabetic retinopathy.

Phenotype	n	Documented DR, n (%)	% of all documented DR	Age, years	Duration, years	eGFR
P1, eGFR ≥ 60 and UACR < 30	2172	233 (10.7)	24.3	59.5	8.2	94.6
P2, eGFR ≥ 60 and UACR ≥ 30	939	313 (33.3)	32.7	62.0	11.4	88.9
P3, eGFR < 60 and UACR < 30	253	52 (20.6)	5.4	73.0	11.9	48.8
P4, eGFR < 60 and UACR ≥ 30	614	360 (58.6)	37.6	71.2	14.9	38.1

Phenotypes use hospital-reported eGFR and admission UACR at KDIGO thresholds. Age, diabetes duration and eGFR are means; eGFR is expressed in mL/min/1.73 m^2^. The adjusted PR for P2 versus P3 was 1.579 (95% CI 1.230–2.147, P = 0.001). Logistic regression with the base covariates was fitted among the 1,192 P2/P3 participants; predictions under each phenotype were standardized over this same sample. The interval uses the 2.5th and 97.5th percentiles of log PR from 1,000 participant-level bootstrap resamples, exponentiated. The two-sided Wald P value uses log PR divided by its bootstrap standard error. eGFR, estimated glomerular filtration rate; PR, prevalence ratio; UACR, urinary albumin-to-creatinine ratio. This exploratory comparison uses nominal inference without correction for multiple analyses.

**Figure 3:**
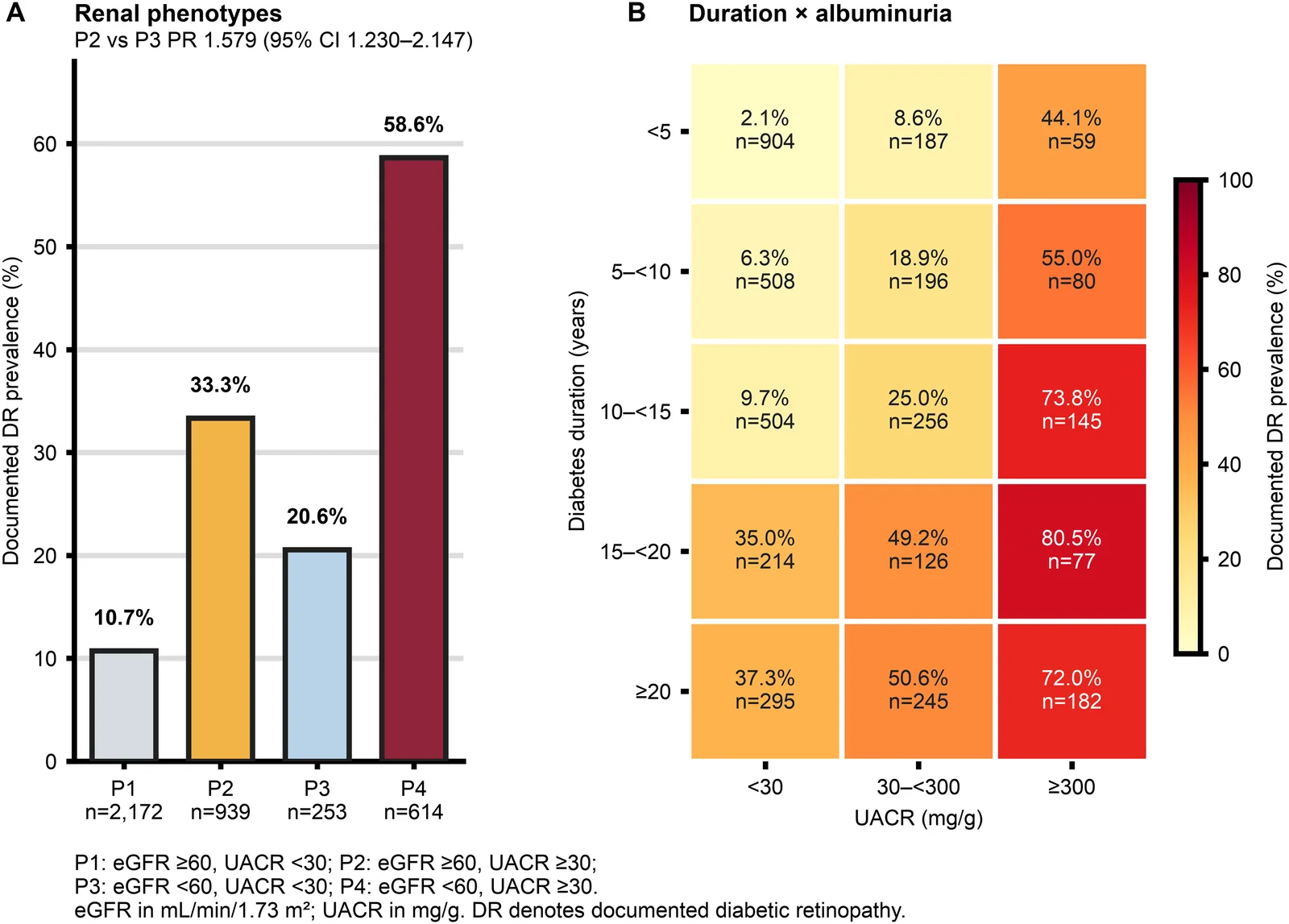
Exploratory renal phenotypes and duration-by-albuminuria prevalence map. Both exploratory panels use the fixed analytic sample (n = 3,978). **(A)** Observed documented DR prevalence in four renal phenotypes defined by eGFR 60 mL/min/1.73 m^2^ and UACR 30 mg/g thresholds. The adjusted P2-versus-P3 comparison is reported in **Table 7**. **(B)** Observed documented DR prevalence by diabetes-duration band (<5, 5–<10, 10–<15, 15–<20 and ≥20 years) and UACR category (<30, 30–<300 and ≥300 mg/g); each cell also shows the number of patients. Bars and cells are descriptive percentages, without confidence intervals. eGFR, estimated glomerular filtration rate; UACR, urinary albumin-to-creatinine ratio; DR, documented diabetic retinopathy.

Documented DR prevalence also increased across UACR categories within diabetes-duration bands (**Figure 3B**), from 2.1% among patients with diabetes duration <5 years and UACR <30 mg/g to 72.0% among those with duration ≥20 years and UACR ≥300 mg/g.

## Discussion

In this single-center inpatient cohort, the choice of renal covariate changed the estimated associations of selected blood markers with documented diabetic retinopathy. Continuous log UACR produced larger proportional reductions in the absolute log PR estimates for serum albumin, fibrinogen and total bilirubin than eGFR, whereas FIB-4 changed little. Thus, eGFR and UACR provided different adjustment information for these associations. Because the study is cross-sectional, these reductions describe changes between fitted models rather than causal mediation.

### Albuminuria and eGFR capture different renal dimensions

eGFR and UACR are often placed side by side in a regression table as if they were alternative measures of the same underlying quantity. Clinically, however, they describe different dimensions of kidney disease. eGFR is a functional measure of filtration, while albuminuria reflects kidney damage and is incorporated independently into chronic kidney disease classification (12). In diabetes, filtration can remain relatively preserved while microvascular injury is already present. This distinction is especially relevant to the retina, where recent clinical and OCTA data have shown a stronger relationship of albuminuria than eGFR with retinopathy severity and retinal non-perfusion (15).

Previous work has mainly compared albuminuria and eGFR as exposures or risk markers for retinopathy. Our analysis addressed a different question: whether choosing one or the other as a renal covariate changes associations attributed to other blood markers. Adding UACR was followed by a substantially larger attenuation of the serum albumin association than adding eGFR, and the mutual-adjustment analysis showed the same asymmetry. With UACR in the model, the eGFR association moved markedly toward the null, whereas adding eGFR to UACR changed the UACR estimate only modestly. This pattern is consistent with evidence that albuminuria captures vascular-risk information in diabetes that is not represented by eGFR alone (14).

The exploratory renal-phenotype analysis provided descriptive context. Patients with preserved eGFR but albuminuria had more documented DR than patients with reduced eGFR but no albuminuria. A meta-analysis found less retinopathy in non-albuminuric than albuminuric diabetic kidney disease overall, but no clear difference when the albuminuric group had preserved eGFR (22).

### What renal adjustment changes in biomarker interpretation

Serum albumin provides the clearest example. Previous observational studies have reported an inverse association between serum albumin and diabetic retinopathy (4, 5). In our base model, the association was also strong and persisted after restriction to eGFR ≥90 mL/min/1.73 m^2^. If renal status were represented only by filtration, this persistence could be interpreted as evidence that the albumin association was largely independent of kidney function. Yet adding log UACR reduced the absolute log PR estimate by approximately three quarters. Restriction to preserved eGFR therefore did not reproduce the change seen when albuminuria was included as a covariate.

Serum albumin is influenced by several physiological processes, including nutritional state and systemic inflammation (6). Fibrinogen, a positive acute-phase reactant, was associated with documented DR in the opposite direction to albumin and had a PR close to one after UACR adjustment, while globulin showed only a weak association that did not persist. Albumin and fibrinogen therefore did not behave as parallel indicators of impaired hepatic synthesis. Their contrasting directions are compatible with the known acute-phase behavior of these proteins (7) and with overlapping renal and inflammatory influences.

After UACR adjustment, a smaller inverse albumin association remained, but its persistence depended on the analysis population and adjustment specification. In the imputed population, the albumin interval included the null after mutual adjustment for eGFR and UACR. The residual association could reflect nutritional or inflammatory information not captured by UACR, residual renal variation from a single urine measurement, or both. Additional adjustment for hemoglobin also weakened the estimate. Together, these findings point to overlapping nutritional, inflammatory and renal influences rather than a purely hepatic or nutritional signal.

The inverse bilirubin association persisted in the complete-case and imputed analyses, including after mutual adjustment for eGFR and UACR (**Table 3** and **Supplementary Table S1**), consistent with observational evidence (8). Beyond antioxidant activity (9), experimental work links bilirubin to reduced adhesion-molecule signaling and leukocyte migration in human endothelial cells and atherosclerosis-prone mice (23). This offers one possible explanation for information not captured by UACR. These non-retinal studies do not establish the mechanism in our cohort, and residual confounding or reverse causation remain plausible. A five-year cohort of men with diabetes reported a U-shaped bilirubin-retinopathy association (24), further arguing against a uniformly protective interpretation.

### Continuous UACR and the role of measurement scale

Continuous and dichotomized UACR produced different adjusted estimates. The 30 mg/g threshold defines a clinically useful albuminuria category (12), but discards information about magnitude and ordering within categories. In our models, binary UACR produced attenuation close to that with eGFR, whereas continuous log UACR produced more than twice that reduction. Because both representations came from the same measurements, this contrast primarily illustrates the effect of model specification and does not establish that the log-linear form is optimal.

The exploratory duration-by-UACR map described variation in documented DR prevalence across categories within duration strata. A single admission UACR also incompletely represents usual albuminuria because of biological variability (25). Classical nondifferential error could weaken adjustment, but its effect on this derived model contrast is uncertain. The observed 38.9-percentage-point difference should therefore not be treated as a lower bound; repeated UACR measurements would be needed to assess this uncertainty.

### FIB-4 and the limits of composite indices

FIB-4 was the exception: its association with documented DR changed little after either eGFR or UACR adjustment. Exploratory component analyses showed an inverse alanine aminotransferase association. This finding requires care because FIB-4 is an arithmetic index developed for liver fibrosis assessment (10). It is now recommended for initial fibrosis risk stratification in adults with type 2 diabetes or prediabetes (11). With age also included as a covariate, the FIB-4 estimate represents variation in the index conditional on age rather than the usual unadjusted clinical score.

Low ALT has been associated with frailty, sarcopenia, disability and poorer outcomes in older populations (26), although whether it reflects these conditions in our inpatient cohort is unknown. A higher FIB-4 value may partly reflect its ALT denominator. Together with FIB-4’s algebraic dependence on age, this limits fibrosis-specific interpretation despite the modest VIFs.

### Implications, strengths and limitations

Using the same analytic sample and base covariates allowed renal-adjustment estimates to be compared without changes in sample composition. The secondary restriction and specification analyses showed how the estimates varied across modeling choices. Laboratory renal measures also made the adjustment definitions explicit. The clinical nephropathy label was strongly associated with documented DR (**Supplementary Table S7**), but its assignment criteria were unavailable; we therefore based the renal comparisons on measured eGFR and UACR.

In the exploratory renal-phenotype analysis, almost one quarter of documented DR cases occurred in patients with eGFR ≥60 mL/min/1.73 m^2^ and UACR <30 mg/g. This finding shows that documented DR also occurred without either laboratory abnormality and should not be interpreted as a screening-performance estimate.

Several limitations shape the interpretation of these findings. The cross-sectional design cannot establish temporal order among albuminuria, blood markers and documented DR. DR = 0 included patients without completed ophthalmological assessment, and examination status and severity were unavailable. If higher UACR prompted more complete ophthalmological evaluation, differential ascertainment could inflate the UACR–DR association and the apparent attenuation after UACR adjustment. The S13 perturbation analysis illustrates the effect of assumed label changes but cannot recover actual examination probabilities. WCC and NLR are imprecise inflammation proxies, and the *post hoc* adjustment cannot exclude residual inflammatory confounding. Treatment, smoking and cardiovascular comorbidity may also confound the observed associations. Equation choice changed eGFR threshold membership; the 2021 sensitivity analysis addressed continuous adjustment in the fixed sample but did not refit threshold-defined subgroup or renal-phenotype analyses.

Missing data are another major constraint. Complete-case analysis excluded 51.2% of the cohort, and UACR measurement was selective. Multiple imputation supported the renal-adjustment comparison for albumin and bilirubin within the observed-albumin population but excluded 739 participants lacking albumin. The common complete-case design therefore improves within-sample model comparability without ensuring representativeness of the full cohort. Finally, this single-center Chinese inpatient cohort may not generalize to community or outpatient populations. Longitudinal studies with repeated UACR, standardized retinal grading and better measurement of inflammation and nutritional status would help clarify how albuminuria, blood markers and retinopathy evolve over time.

## Conclusions

In this single-center cohort of adults with type 2 diabetes, continuous log UACR adjustment reduced the absolute log PR estimates for serum albumin, fibrinogen and total bilirubin more than eGFR adjustment. FIB-4 changed little. eGFR and UACR therefore provided different adjustment information for these specific blood-marker associations with documented DR. Dichotomizing UACR yielded smaller reductions. These findings are descriptive and specific to the examined models and population; the exploratory FIB-4 component and renal-phenotype analyses require confirmation.

## Data Availability

The datasets presented in this article are not readily available because they contain clinical information that could compromise individual privacy, and access is subject to approval by the institutional ethics committee. Requests to access the datasets should be directed to Long Pang, 1907757091@qq.com.

## Supplementary material

Supplementary Table S1–S14 Additional analyses, diagnostics, sensitivity analyses and outcome perturbation analysis.
