# Supplementary material for: UACR adjustment attenuates associations of selected routine blood markers with documented diabetic retinopathy more than eGFR adjustment: a cross-sectional study in type 2 diabetes
Source: Front Endocrinol (Lausanne). 2026 Oct 27;17:1983060. doi: 10.3389/fendo.2026.1983060 (PMC13747845; doi:10.3389/fendo.2026.1983060)
Supplement: Supplementary file 1 [file SupplementaryFile1.docx]

# Supplementary Material 1. Supplementary Tables S1-S14

DR denotes documented diabetic retinopathy throughout. DR=0 means no documented DR and includes patients without completed ophthalmological assessment.

**Table S1 Multiple imputation compared with complete-case analysis**

A. M3 comparison with corrected pooling. All estimates are prevalence ratios under M3 (base covariates + log UACR), per 1-standard-deviation increase. Panels B–E report the expanded sensitivity analysis.

| Marker | Complete case (n = 3978) | Multiple imputation (n = 7420) | t | df | P | FMI |
| --- | --- | --- | --- | --- | --- | --- |
| Serum albumin | 0.907 (0.861–0.954)　P < 0.001 | 0.955 (0.915–0.997) | -2.09 | 2086 | 0.037 | 0.067 |
| Fibrinogen | 0.994 (0.940–1.045)　P = 0.812 | 1.007 (0.966–1.050) | 0.31 | 1488 | 0.755 | 0.079 |
| Total bilirubin | 0.915 (0.866–0.964)　P < 0.001 | 0.923 (0.887–0.961) | -3.93 | 4228 | <0.001 | 0.047 |
| FIB-4 index | 1.084 (1.027–1.139)　P = 0.003 | 1.076 (1.030–1.124) | 3.29 | 1584 | 0.001 | 0.077 |
| Globulin (non-albumin protein control) | 0.975 (0.931–1.019)　P = 0.283 | 0.997 (0.958–1.038) | -0.14 | 2496 | 0.890 | 0.061 |

Statistical methods

**Multiple imputation by chained equations** (MICE) was used. Entry required observed DR coding, serum albumin, age and sex (n = 7,420). Documented DR coding, age and sex were complete in all 8,159 records; 739 participants lacked albumin and were outside the imputation sample. This restriction kept albumin observed while imputing other incomplete analysis variables. UACR required imputation in 2,110 of 7,420 participants (28.4%). The imputation model included DR coding, albumin, total bilirubin, fibrinogen, FIB-4, globulin, UACR, eGFR, age, sex, diabetes duration, HbA1c, systolic blood pressure and BMI. Total bilirubin, fibrinogen, FIB-4 and UACR were natural-log transformed before imputation.

Iteration settings were 20 burn-in updates followed by one retained dataset every 10 updates, for 10 datasets in total. The random seed was 20260801. Observed values were preserved. All marker contrasts used the standard deviation from the original 3,978 complete cases. Each completed dataset was analyzed using the same marginal-standardization definition as the main analysis.

Model log prevalence ratios were combined using Rubin's rules. Within-imputation variances were estimated from 1,000 bootstrap row resamples per completed dataset; pooled intervals below are t-based, not bootstrap percentile intervals. Total variance was T = W + (1 + 1/m)B, where W is the mean within-imputation variance and B the between-imputation variance. With lambda = (1 + 1/m)B/T, degrees of freedom were (m − 1)/lambda². Inference used the t distribution. FMI was lambda + (1 − lambda) × 2/(df + 3). These are classic Rubin degrees of freedom, without the Barnard–Rubin adjustment. The corrected FMI range in panel A is 0.047–0.079. Low FMI does not establish the validity of the missing-at-random assumption.

Complete-case analysis remains primary to present the four models in a common population with observed exposures and adjustment variables. A common sample can also be maintained after imputation, so this design feature does not establish that complete-case analysis is unbiased or superior. The imputation analysis is a sensitivity assessment within the observed-albumin population.

UACR measurement was selective (Table S5). Imputation assumes that missingness is at random conditional on observed variables and that the imputation model is adequate. It cannot exclude selection bias or differences in documented DR ascertainment. Among the 739 participants outside the imputation sample, 68 (9.2%) had documented DR, 734 lacked bilirubin and 676 lacked a calculable FIB-4. Their missingness pattern and the model-specific uncertainty are reported below.

**B. Four-model multiple-imputation estimates**

All models use the same 7,420 participants and 10 imputed datasets. M1 includes the base covariates; M2 adds eGFR, M3 adds log UACR, and M4 adds both. PRs and 95% CIs use the corrected pooling specification above. Total bilirubin, fibrinogen and FIB-4 are natural-log transformed. P values are unadjusted.

| **Marker** | **Model** | **PR (95% CI)** | **t** | **df** | **P** | **FMI** |
| --- | --- | --- | --- | --- | --- | --- |
| Serum albumin | M1 | 0.729 (0.698–0.762) | -14.00 | 27303 | <0.001 | 0.018 |
| Serum albumin | M2 | 0.827 (0.793–0.863) | -8.75 | 30348 | <0.001 | 0.017 |
| Serum albumin | M3 | 0.955 (0.915–0.997) | -2.09 | 2086 | 0.037 | 0.067 |
| Serum albumin | M4 | 0.969 (0.929–1.010) | -1.47 | 4962 | 0.141 | 0.043 |
| Total bilirubin | M1 | 0.764 (0.729–0.801) | -11.23 | 16104 | <0.001 | 0.024 |
| Total bilirubin | M2 | 0.869 (0.832–0.907) | -6.45 | 18880 | <0.001 | 0.022 |
| Total bilirubin | M3 | 0.923 (0.887–0.961) | -3.93 | 4228 | <0.001 | 0.047 |
| Total bilirubin | M4 | 0.942 (0.906–0.980) | -2.96 | 9084 | 0.003 | 0.032 |
| Fibrinogen | M1 | 1.263 (1.218–1.310) | 12.56 | 6313 | <0.001 | 0.038 |
| Fibrinogen | M2 | 1.126 (1.082–1.171) | 5.91 | 7420 | <0.001 | 0.035 |
| Fibrinogen | M3 | 1.007 (0.966–1.050) | 0.31 | 1488 | 0.755 | 0.079 |
| Fibrinogen | M4 | 0.988 (0.948–1.031) | -0.55 | 3145 | 0.581 | 0.054 |
| FIB-4 index | M1 | 1.091 (1.040–1.145) | 3.56 | 20709 | <0.001 | 0.021 |
| FIB-4 index | M2 | 1.082 (1.032–1.135) | 3.25 | 7070 | 0.001 | 0.036 |
| FIB-4 index | M3 | 1.076 (1.030–1.124) | 3.29 | 1584 | 0.001 | 0.077 |
| FIB-4 index | M4 | 1.075 (1.029–1.123) | 3.23 | 1658 | 0.001 | 0.075 |
| Globulin | M1 | 1.095 (1.050–1.141) | 4.27 | 246495 | <0.001 | 0.006 |
| Globulin | M2 | 1.010 (0.969–1.053) | 0.47 | 381150 | 0.639 | 0.005 |
| Globulin | M3 | 0.997 (0.958–1.038) | -0.14 | 2496 | 0.890 | 0.061 |
| Globulin | M4 | 0.981 (0.942–1.021) | -0.96 | 4888 | 0.339 | 0.043 |

**C. Paired comparison of UACR and eGFR adjustment**

Within each completed dataset, model-3-minus-model-2 log PR differences were calculated using 1,000 shared bootstrap row resamples. Their variances preserve the covariance of model estimates. Rubin pooling was applied to these scalar differences, then exponentiated to obtain PR(M3)/PR(M2). Ratios must be interpreted alongside the underlying PRs; values above one do not universally mean greater attenuation. These revision-stage sensitivity P values are unadjusted.

| **Marker** | **PR(M3)/PR(M2) (95% CI)** | **t** | **df** | **P** | **FMI** |
| --- | --- | --- | --- | --- | --- |
| Serum albumin | 1.155 (1.127–1.183) | 11.74 | 378 | <0.001 | 0.159 |
| Total bilirubin | 1.063 (1.040–1.086) | 5.63 | 627 | <0.001 | 0.123 |
| Fibrinogen | 0.894 (0.874–0.916) | -9.38 | 244 | <0.001 | 0.199 |
| FIB-4 index | 0.994 (0.975–1.013) | -0.62 | 550 | 0.535 | 0.131 |
| Globulin | 0.987 (0.969–1.006) | -1.32 | 246 | 0.187 | 0.198 |

**D. Additional attenuation estimates**

In each completed dataset, attenuation was 100 × [1 − |log PR(adjusted)|/|log PR(M1)|]. Estimates were then pooled on the percentage-point scale, using paired bootstrap within-imputation variances. The intervals are approximate t-based intervals. They describe changes after adjustment, not causal mediation fractions. Quantitative interpretation is limited to albumin and bilirubin, as in the main analysis.

| **Marker** | **eGFR attenuation % (95% CI)** | **UACR attenuation % (95% CI)** | **Difference in percentage points (95% CI)** |
| --- | --- | --- | --- |
| Serum albumin | 39.9 (33.0–46.9) | 85.5 (73.8–97.3) | 45.6 (36.4–54.8) |
| Total bilirubin | 47.6 (38.8–56.3) | 70.2 (59.1–81.4) | 22.7 (14.5–30.8) |

The M1 bootstrap log PR did not cross or approach zero for albumin or bilirubin. Albumin M3 crossed zero in 126 of 10,000 diagnostic draws, so its absolute-value attenuation interval requires caution; the smooth log PR contrast in panel C is the principal direct comparison. These draws combine diagnostic counts across imputations, not independent participants. Nonlinear attenuation inference for fibrinogen, FIB-4 and globulin is not reported.

**E. Missingness and analysis populations**

The 7,420-participant imputation sample contains all 3,978 complete cases and 3,442 additional participants. Counts below overlap across variables and must not be summed as separate exclusions. Documented DR, age and sex had no missing values; this does not establish universal ophthalmological assessment.

| **Variable** | **Missing in full cohort (n = 8,159)** | **Missing before MI (n = 7,420)** | **Missing outside MI (n = 739)** |
| --- | --- | --- | --- |
| DR coding | 0 (0.0%) | 0 (0.0%) | 0 (0.0%) |
| Serum albumin | 739 (9.1%) | 0 (0.0%) | 739 (100.0%) |
| Total bilirubin | 768 (9.4%) | 34 (0.5%) | 734 (99.3%) |
| Fibrinogen | 346 (4.2%) | 170 (2.3%) | 176 (23.8%) |
| FIB-4 | 991 (12.1%) | 315 (4.2%) | 676 (91.5%) |
| Globulin | 748 (9.2%) | 9 (0.1%) | 739 (100.0%) |
| UACR | 2355 (28.9%) | 2110 (28.4%) | 245 (33.2%) |
| eGFR | 369 (4.5%) | 141 (1.9%) | 228 (30.9%) |
| Age | 0 (0.0%) | 0 (0.0%) | 0 (0.0%) |
| Sex | 0 (0.0%) | 0 (0.0%) | 0 (0.0%) |
| Diabetes duration | 757 (9.3%) | 641 (8.6%) | 116 (15.7%) |
| HbA1c | 1158 (14.2%) | 754 (10.2%) | 404 (54.7%) |
| Systolic blood pressure | 90 (1.1%) | 79 (1.1%) | 11 (1.5%) |
| BMI | 349 (4.3%) | 322 (4.3%) | 27 (3.7%) |

The pooled degrees-of-freedom, confidence-interval and FMI calculations in S1A were corrected; the M3 point estimates are unchanged. Panels B–E report the expanded four-model analysis and paired comparisons.

**Table S2 Sensitivity analyses for serum albumin**

All estimates are expressed in **a single standard-deviation unit** (4.230 g/L, from the main analytic sample).

| Analysis | n | M1 base covariates | z | M3 + log(UACR) | z | P |
| --- | --- | --- | --- | --- | --- | --- |
| Main analysis (all) | 3978 | 0.694 (0.655–0.731) | −13.43 | 0.907 (0.861–0.954) | −3.61 | <0.001 |
| Excluding hypoalbuminemia (ALB ≥ 35 g/L) | 3631 | 0.675 (0.616–0.739) | −8.22 | **0.836 (0.769–0.905)** | −4.33 | **<0.001** |
| Excluding marked transaminase elevation (ALT/AST ≤ 150 U/L) | 3955 | 0.693 (0.655–0.733) | −12.29 | 0.905 (0.856–0.955) | −3.63 | <0.001 |
| Men only | 2168 | 0.675 (0.621–0.729) | −9.83 | 0.922 (0.854–0.993) | −2.08 | 0.038 |
| Women only | 1810 | 0.715 (0.657–0.772) | −8.33 | 0.894 (0.826–0.965) | −2.87 | 0.004 |
| Statin non-users only | 3203 | 0.694 (0.648–0.739) | −10.87 | 0.906 (0.851–0.963) | −3.08 | 0.002 |
| Restricted to eGFR ≥ 60 | 3111 | 0.753 (0.688–0.824) | −6.06 | 0.905 (0.832–0.982) | −2.33 | 0.020 |
| Excluding macroalbuminuria (UACR < 300 mg/g) | 3435 | 0.825 (0.757–0.905) | −4.21 | **0.878 (0.805–0.958)** | −2.98 | **0.003** |
| Additionally adjusted for hemoglobin | 3976 | 0.788 (0.743–0.836) | −7.99 | 0.949 (0.897–1.000) | −1.85 | 0.064 |

Statistical methods

Effect measure and test statistic as in main-text Table 2 (prevalence ratios by marginal standardization; z and P from the bootstrap standard error on the log scale). In the sex-stratified analyses sex is no longer included as a covariate.

On the standardization scale. The standard deviation of serum albumin differs across subsets (4.230 g/L in the main analysis, 3.208 after excluding hypoalbuminemia, 3.803 after restriction to eGFR ≥ 60). Standardising within each subset would make “per 1 standard deviation” represent a different absolute change in each row, so that rows could not be compared directly. This table uses the standard deviation of the main analytic sample (4.230 g/L) throughout, so that all rows correspond to the same absolute change and are horizontally comparable.

These subset analyses should be interpreted cautiously because restriction changes both the exposure distribution and the covariate structure; they were intended as robustness checks rather than formal tests of effect modification.

The UACR-adjusted serum albumin PR remained below one in all eight sensitivity analyses. Its 95% confidence interval excluded one in seven analyses; after additional hemoglobin adjustment, the interval included one (PR 0.949, 95% CI 0.897–1.000; P = 0.064). These analyses support the direction of the main finding but should not be read as formal effect-modification tests.

**Table S3 Renal mutual adjustment and FIB-4 collinearity diagnostics**

**A. Mutual adjustment of eGFR and UACR**

| Renal index | Entered alone | z | After mutual adjustment | z | Association attenuation |
| --- | --- | --- | --- | --- | --- |
| eGFR (per +10 mL/min/1.73 m²) | 0.836 (0.820–0.854) | −16.92 | 0.951 (0.930–0.974) | −4.14 | **71.9%** |
| log(UACR) (per 1 SD) | 1.652 (1.596–1.708) | 28.88 | 1.571 (1.504–1.641) | 20.23 | **10.0%** |

Statistical methods

Both models were fitted in the fixed analytic sample (n = 3978) with adjustment for age, sex, diabetes duration, glycated hemoglobin, systolic blood pressure and body mass index.

The marginal prevalence ratios were estimated by standardizing predictions over the same analytic population. Changes after mutual adjustment remain descriptive and can reflect several features of the data and model; they do not isolate confounding, mediation or a causal proportion explained.

Collinearity: variance inflation factors for eGFR and log(UACR) were 2.19 and 1.57; their Pearson correlation was −0.491 (computed on the log scale; the correlation between eGFR and UACR on the original scale is −0.418).

**B. Predictor-design VIFs for the FIB-4 models**

The original 3,978 complete cases (958 with documented DR) were retained. Each model included age in years, sex, diabetes duration, HbA1c, systolic blood pressure, BMI and natural-log FIB-4; M2 added reported eGFR, M3 added log UACR and M4 added both. These revision-stage diagnostics used the primary model designs and did not change the fitted associations.

| **Predictor** | **M1** | **M2** | **M3** | **M4** |
| --- | --- | --- | --- | --- |
| Age | 1.922 | 2.415 | 1.923 | 2.535 |
| Sex | 1.048 | 1.065 | 1.052 | 1.065 |
| Diabetes duration | 1.250 | 1.271 | 1.326 | 1.326 |
| HbA1c | 1.028 | 1.039 | 1.049 | 1.088 |
| Systolic blood pressure | 1.086 | 1.101 | 1.163 | 1.163 |
| BMI | 1.060 | 1.079 | 1.063 | 1.080 |
| log(FIB-4) | 1.546 | 1.546 | 1.546 | 1.546 |
| eGFR | — | 1.678 | — | 2.187 |
| log(UACR) | — | — | 1.204 | 1.569 |

VIF = 1/(1 − R²), where R² comes from an unweighted auxiliary linear regression of a predictor on all remaining predictors with an intercept. The intercept VIF is not reported. Dashes indicate predictors not included in that model. Values are unchanged by linear standardization.

All four design matrices were full rank. Age and log(FIB-4) had Pearson r = 0.588; the maximum VIF across all models was 2.535. These design diagnostics did not suggest marked linear collinearity, but they do not estimate the variance inflation of marginal PRs or establish absence of confounding. FIB-4 retains its algebraic dependence on age, and its fitted association remains conditional on the modeled age adjustment.

Table S4 Exploratory associations of FIB-4 components (full version of Table 6)

| Component | Entered alone | z | All three entered together | z |
| --- | --- | --- | --- | --- |
| Aspartate aminotransferase (log) | 0.983 (0.926–1.037) | −0.61 | 1.195 (1.106–1.292) | 4.31 |
| Alanine aminotransferase (log) | 0.876 (0.821–0.930) | −4.29 | 0.747 (0.679–0.822) | −5.97 |
| Platelet count | 0.982 (0.929–1.032) | −0.67 | 0.974 (0.920–1.025) | −0.94 |
| FIB-4 index (composite, reference) | 1.103 (1.039–1.165) | 3.26 | — | — |

Statistical methods as in the footnote to main-text Table 6.

**Table S5 Included versus excluded participants, and selection into UACR measurement**

| Variable | Included (n = 3978) | Excluded (n = 4181) | P |
| --- | --- | --- | --- |
| Age, years | 62.76 ± 12.98 | 61.89 ± 13.91 | 0.004 |
| Diabetes duration, years | 10.19 ± 7.97 | 9.13 ± 7.90 | <0.001 |
| Glycated hemoglobin, % | 8.81 ± 2.30 | 8.89 ± 2.55 | 0.199 |
| Systolic blood pressure, mmHg | 135.34 ± 20.06 | 132.22 ± 18.91 | <0.001 |
| Body mass index, kg/m² | 24.20 ± 3.84 | 24.09 ± 3.90 | 0.221 |
| eGFR, mL/min/1.73 m² | 81.62 ± 26.77 | 84.96 ± 25.17 | <0.001 |
| Documented DR, n (%) | 958 (24.1) | 661 (15.8) | <0.001 |

**Selection into UACR measurement.** After adjustment for age, sex, systolic blood pressure, eGFR and diabetes duration (logistic regression with “UACR measured” as the outcome), documented DR was associated with higher odds of UACR measurement (OR 1.357; P < 0.001). UACR was measured in 5804 of the 8159 participants (71.1%).

Statistical methods

Continuous variables were compared using the independent-samples t test with Welch’s correction and categorical variables using the Pearson chi-squared test without continuity correction, as elsewhere.

Missing-data note. Excluded participants had a lower prevalence of documented DR than included participants (15.8% vs 24.1%), but the direction of selection bias cannot be determined from observed data alone. The multiple-imputation analysis (Table S1) included a larger sample and gave similar qualitative conclusions.

On selection into UACR measurement: UACR testing was associated with documented DR and other observed clinical factors, so complete-case analyses may be affected by selection. This is one reason multiple imputation is reported as a sensitivity analysis.

**Table S6 Two markers not carried forward into the primary analysis**

**Note on samples.** Neither prothrombin time nor gamma-glutamyl transferase belongs to the variable set defining the fixed analytic sample, so the analyses in this table are drawn from the **full cohort of 8159** among those with the relevant marker and covariates available. The sample sizes therefore differ from the 3978 used in the main-text tables and are not directly comparable. The purpose of this table is to document the reasons for exclusion, not to provide effect estimates alongside the primary analysis.

**S6a Prothrombin time**

The international normalized ratio is calculated from prothrombin time (PT), the assay-specific mean normal PT and the international sensitivity index (ISI): INR = (PT/mean normal PT)^ISI. A monotone relation therefore does not by itself imply a constant PT/INR ratio.

| Quantity | Value |
| --- | --- |
| Coefficient of variation of prothrombin time ÷ international normalized ratio | 0.085 |
| Proportion of records with a ratio between 11.0 and 11.8 | 57.4% |
| Spearman correlation, all records | 0.548 |
| Spearman correlation, records with a consistent ratio | 0.975 |

| Subset | n | Prevalence ratio for prothrombin time | P |
| --- | --- | --- | --- |
| Consistent ratio (11.0–11.8) | 3370 | 1.075 (1.013–1.145) | 0.018 |
| Aberrant ratio | 2497 | 1.112 (1.050–1.175) | <0.001 |

Rationale. If PT and INR derive from the same specimen and are calculated with stable calibration, a strong monotone relation is expected. The observed overall rank correlation was 0.548, compared with 0.975 among records with a relatively consistent ratio. These discrepancies raised concerns about measurement comparability, but the available data could not distinguish calibration or reagent differences, mismatched sampling, or data-linkage problems. Because measurement reliability could not be established, PT was not carried forward into the primary analyses.

**S6b Gamma-glutamyl transferase (participants with eGFR ≥ 60)**

| Subgroup | n | Prevalence ratio | P |
| --- | --- | --- | --- |
| All | 4650 | 0.909 (0.844–0.973) | 0.009 |
| Never drinkers | 1874 | 0.972 (0.880–1.064) | 0.557 |
| Former or current drinkers | 519 | 0.924 (0.682–1.144) | 0.553 |
| Drinking history not recorded | 2257 | 0.785 (0.683–0.880) | <0.001 |

Rationale. The inverse estimate was strongest in the subgroup with unrecorded drinking history. Confidence intervals included one in the two subgroups with recorded history, but this does not establish absence of association or a between-subgroup difference. This pattern raises concern about record completeness and residual confounding without establishing their cause. Gamma-glutamyl transferase was not carried forward into the primary marker set.

Statistical methods for S6a and S6b. Logistic marginal standardization adjusted for age, sex, diabetes duration, HbA1c, systolic blood pressure and BMI. Contrasts increased natural-log marker values by one subgroup-specific SD. The 95% intervals are exponentiated 2.5th/97.5th percentiles of log PR from 1,000 participant-level bootstrap resamples. Two-sided Wald P values use log PR divided by its bootstrap standard error. No between-subgroup interaction test was performed.

**Table S7 Association between the clinical nephropathy diagnosis and documented DR**

| Group | n | Documented DR prevalence |
| --- | --- | --- |
| Diabetic nephropathy diagnosis positive | 836 | 82.4% |
| Diabetic nephropathy diagnosis negative | 7323 | 12.7% |

**The recorded clinical nephropathy diagnosis was strongly associated with documented DR.** This descriptive disparity does not identify the clinical criteria used to assign the diagnosis and cannot by itself establish diagnostic circularity. The analysis compares laboratory-defined **renal covariates,** eGFR and UACR, rather than the clinical diagnostic label. This choice does not eliminate shared disease burden, residual confounding or differential ascertainment.

**Table S8 Post hoc analysis of the acute-phase response (n = 3974)**

| Marker | M1 base covariates | M1 + inflammation markers | M3 + inflammation markers |
| --- | --- | --- | --- |
| Serum albumin | 0.695 (0.655–0.732) | 0.719 (0.679–0.758) | 0.914 (0.866–0.965)　P = 0.002 |
| Fibrinogen | 1.243 (1.190–1.305) | 1.217 (1.158–1.285) | 0.998 (0.945–1.057)　P = 0.956 |
| Total bilirubin | 0.759 (0.716–0.805) | 0.768 (0.725–0.812) | 0.911 (0.868–0.959)　P < 0.001 |
| FIB-4 index | 1.105 (1.043–1.169) | 1.133 (1.066–1.205) | 1.069 (1.008–1.130)　P = 0.022 |
| Alanine aminotransferase (log) | 0.874 (0.820–0.926) | 0.886 (0.834–0.938) | **0.945 (0.898–0.989)**　P = 0.019 |
| Globulin (non-albumin protein control) | 1.066 (1.014–1.122) | 1.061 (1.009–1.116) | 0.983 (0.940–1.031)　P = 0.479 |

Statistical methods

C-reactive protein and procalcitonin were unavailable in the analysis dataset. White cell count (WCC; stored as WBC) and the neutrophil-to-lymphocyte ratio (NLR), both natural-log transformed and standardized, were added to M1 and M3 as inflammation proxies. Four of the 3,978 complete cases lacked WCC or NLR, leaving the same 3,974 participants for all models in Table S8. Marker contrasts retained the standard deviations from the original complete-case sample.

This post hoc analysis examined whether additional adjustment for inflammation proxies changed the associations of serum albumin, a negative acute-phase reactant, and fibrinogen, a positive acute-phase reactant. It cannot distinguish acute illness from protein loss or establish a mechanism.

Within the same 3,974 participants, adding WCC and NLR to M1 moved the serum albumin PR from 0.695 to 0.719 and the fibrinogen PR from 1.243 to 1.217. With log UACR and both inflammation proxies included, the serum albumin PR was 0.914 (95% CI 0.866–0.965; P = 0.002) and the fibrinogen PR was 0.998 (0.945–1.057; P = 0.956). The albumin association remained inverse in this specification.

With log UACR and both inflammation proxies included, the FIB-4 PR was 1.069 (1.008–1.130; P = 0.022). The primary M3 estimate of 1.084 used 3,978 participants, so its comparison with this 3,974-participant estimate also involves a small change in sample composition. The component analysis is reported in main-text Table 6.

Alanine aminotransferase remained inversely associated with documented DR after simultaneous adjustment for log UACR and both inflammation proxies (PR 0.945, 95% CI 0.898–0.989; P = 0.019). This separate component association does not by itself identify the source or mechanism of the composite FIB-4 association.

The M1 value for alanine aminotransferase in this table is 0.874 (0.820-0.926), differing slightly from the 0.876 (0.821-0.930) in the "entered alone" column of main-text Table 6 because this table uses n = 3974 after excluding four participants with missing inflammation markers.

This exploratory post hoc analysis is described under Sensitivity and exploratory analyses in the Methods. It did not fit inflammation-adjusted M2 or M4 models or directly re-estimate the UACR-versus-eGFR attenuation difference. No inference about that difference after inflammatory adjustment is made from Table S8.

WCC and NLR are imprecise inflammation proxies that cannot replace C-reactive protein; residual inflammatory confounding remains possible despite this adjustment.

**Table S9 Unadjusted and adjusted prevalence ratios (fixed analytic sample, n = 3978)**

| Marker | Unadjusted | M1, adjusted for base covariates |
| --- | --- | --- |
| Serum albumin | 0.617 (0.584–0.649) | 0.694 (0.655–0.731) |
| Total bilirubin | 0.692 (0.650–0.736) | 0.759 (0.714–0.802) |
| Fibrinogen | 1.343 (1.282–1.402) | 1.243 (1.186–1.297) |
| FIB-4 index | 1.278 (1.220–1.339) | 1.103 (1.039–1.165) |
| Globulin (non-albumin protein control) | 1.092 (1.031–1.145) | 1.065 (1.010–1.118) |
| eGFR (per +10 mL/min/1.73 m²) | 0.807 (0.795–0.820) | 0.836 (0.820–0.854) |
| log(UACR) (per 1 SD) | 1.798 (1.750–1.849) | 1.652 (1.596–1.708) |

Statistical methods

This table reports unadjusted alongside adjusted estimates as required by STROBE item 16(a). Both columns are fitted in the same fixed analytic sample using the same standardization units (taken from that sample); all estimates are per 1-standard-deviation increase except eGFR, which is per 10 mL/min/1.73 m². The “unadjusted” column contains models with the exposure and an intercept only.

M1 is adjusted for age, sex, diabetes duration, glycated hemoglobin, systolic blood pressure and BMI. These represent demographic background, disease duration, recent glycemic status, blood pressure and body size, providing the common adjustment set described in the main Methods.

All four primary exposure estimates move toward the null after base adjustment and retain **their directions. Agreement in direction does not establish insensitivity to covariate selection or absence of residual confounding. Table S2 reports selected alternative specifications and restrictions.**

Table S10. Choice of estimator: log-link Poisson versus marginal standardization

**A. Out-of-range fitted probabilities under the log-link Poisson model (exposure: serum albumin)**

| Model | Observations with fitted probability > 1 | Proportion | Largest fitted probability, Poisson | Largest fitted probability, marginal standardization | Mean serum albumin among these patients (g/L) | Documented DR prevalence among them | Median UACR among them (mg/g) |
| --- | --- | --- | --- | --- | --- | --- | --- |
| M1 | 48 | 1.21% | 3.997 | 0.987 | 32.2 | 72.9% | 690.7 |
| M2 | 75 | 1.89% | 3.568 | 0.983 | 33.7 | 78.7% | 2047.5 |
| M3 | 106 | 2.66% | 3.799 | 0.99 | 34.6 | 80.2% | 2393.1 |
| M4 | 109 | 2.74% | 3.672 | 0.99 | 34.8 | 78.9% | 2357.9 |

**B. Prevalence ratio per 1-SD increase in serum albumin, both estimators side by side**

| Model | Log-link Poisson (robust variance) | Marginal standardization (primary analysis) |
| --- | --- | --- |
| M1 | 0.735 (0.702–0.770), P < 0.001 | 0.694 (0.655–0.731), P < 0.001 |
| M2 | 0.821 (0.783–0.861), P < 0.001 | 0.787 (0.745–0.828), P < 0.001 |
| M3 | 0.962 (0.915–1.011), P = 0.125 | 0.907 (0.861–0.954), P < 0.001 |
| M4 | 0.967 (0.920–1.016), P = 0.180 | 0.919 (0.871–0.966), P = 0.002 |

Statistical methods

A log link can return fitted probabilities above 1. In these data, out-of-range fitted values increased with adjustment depth (48, 75, 106 and 109 observations across M1-M4) and were concentrated among patients with low serum albumin, high documented DR prevalence and high UACR.

Log-binomial regression did not converge. The primary analysis therefore used marginal standardization from a logistic outcome model: fitted probabilities remained within bounds, and standardized prevalences were contrasted to report prevalence ratios rather than conditional odds ratios.

Panel B compares marginal standardization with log-link Poisson estimates. The estimates agree in direction and are close in magnitude, with the main difference appearing after log(UACR) adjustment, where the Poisson model had the most out-of-range fitted values. For serum albumin in M3, confidence-interval inclusion of one differed between estimators; directional agreement does not imply identical inference.

Table S11. Association attenuation after renal adjustment, with bootstrap confidence intervals

| Marker | Attenuated after eGFR adjustment | Attenuated after log(UACR) adjustment | Difference (percentage points) |
| --- | --- | --- | --- |
| Serum albumin | 34.3% (26.9–42.3) | 73.3% (63.0–85.8) | 38.9 (30.7–49.0) |
| Total bilirubin | 43.6% (34.3–54.7) | 67.9% (56.2–83.5) | 24.3 (15.8–34.5) |
| Fibrinogen | 50.9% (39.1–70.2) | 97.1% (65.5–99.6) | 46.2 (−0.9–54.8) |
| FIB-4 index | 12.6% (-6.0–40.2) | 18.4% (-12.1–48.7) | 5.7 (-25.9–30.8) |

Statistical methods

Attenuation is the proportional reduction in the absolute log PR estimate, expressed as a percentage, 100 × [1 − |log PR(Mk)|/|log PR(M1)|]. The 95% intervals are the 2.5th and 97.5th percentiles on the percentage-point scale from 1,000 non-parametric bootstrap row resamples. M1, M2 and M3 were refitted within each shared resample, preserving their dependence. No hypothesis-test P values are reported.

For serum albumin and total bilirubin, the attenuation-difference intervals exclude zero. The fibrinogen and FIB-4 attenuation results are exploratory. In 419 of 1,000 resamples, the fibrinogen M3 log PR had the opposite sign to its point estimate. The FIB-4 M1 log PR changed sign in one resample and approached zero (minimum absolute value 0.000522), making division by this quantity unstable. Their attenuation percentages and intervals should therefore be read cautiously as descriptive model contrasts.

The main text quantifies attenuation only for serum albumin and total bilirubin. For fibrinogen, the M3 PR is 0.994 (95% CI 0.940–1.045); this interval includes one and does not demonstrate absence of association. Its paired M3/M2 PR ratio is 0.893 (0.870–0.916), using 2,000 shared bootstrap resamples and an exponentiated percentile interval on the log-ratio scale. FIB-4 interpretation relies on its individual model estimates, not on its unstable attenuation percentage. These comparisons do not establish a causal role for UACR.

Table S12. Effect of renal-variable specification on association attenuation

Prevalence ratio per 1-SD increase in serum albumin, fixed analytic sample n = 3978.

| Model specification | Serum albumin PR (95% CI) | M1 log-PR attenuated |
| --- | --- | --- |
| M1 base covariates (no renal index) | 0.694 (0.655–0.731) | 0.0% |
| + binary albuminuria (UACR ≥ 30 mg/g) | 0.782 (0.741–0.821) | 32.5% |
| + binary renal index (UACR ≥ 30 or eGFR < 60) | 0.774 (0.734–0.814) | 29.9% |
| + eGFR (continuous) | 0.787 (0.745–0.828) | 34.3% |
| **+ log(UACR) (continuous)** | 0.907 (0.861–0.954) | 73.3% |

Statistical methods

The underlying renal measurements and analytic sample are identical across specifications; only their model representation changes. Binary albuminuria groups all UACR values below 30 mg/g together and all values at or above 30 mg/g together, discarding variation within those categories.

The proportional reduction in the absolute serum-albumin log PR estimate was 32.5% with binary albuminuria and 73.3% with continuous log UACR. The binary composite produced similar attenuation to binary albuminuria alone.

The weaker adjustment with binary UACR may partly reflect information loss through dichotomization. The comparison does not establish that binary and continuous UACR are different biological constructs, or that the chosen continuous functional form is optimal.

The binary composite is derived from laboratory UACR and eGFR thresholds and is not a clinical diagnosis. The recorded clinical nephropathy diagnosis agreed poorly with this laboratory variable, with Cohen’s kappa = 0.270 (n = 5567); 26.8% of patients meeting the laboratory criteria carried the diagnosis. Documented DR prevalence was 82.4% among those carrying the diagnosis and 12.7% among those not. These observations establish association and discordance, not how clinicians assigned the diagnosis (Table S7). These findings are dataset-specific.

**Table S13. Forward perturbation sensitivity analysis for outcome misclassification**

A. Scope and probability-scale identity. The analysis outcome is documented DR; DR=0 includes unassessed patients without a DR discharge diagnosis. The frequency and determinants of incomplete assessment could not be estimated from the analysis dataset. Under constant sensitivity Se and specificity 1, the observed outcome probability is Se multiplied by the true probability. The factor Se cancels in a ratio of standardized probabilities when the same weights are used. This identity does not guarantee invariance after refitting an ordinary logistic model, because Se multiplied by a logistic probability is generally not itself logistic. The unperturbed serum albumin model-3 PR was 0.907. No empirical bias correction or recovery of true DR status is implied.

B. Perturbation of observed outcome labels. The table reports 11 assumed scenarios, with 200 realizations per scenario in the fixed complete-case sample (n = 3,978). Sensitivity pairs give the probabilities of retaining an observed DR=1 label for UACR <30 and ≥30 mg/g, respectively. Equal values specify non-differential perturbation; unequal values specify differential sensitivity by albuminuria. Specificity is the probability of retaining an observed DR=0 label. These parameters describe additional perturbation of recorded labels and are not estimates of clinical diagnostic performance.

| **Sensitivity low / high UACR** | **Specificity** | **Mean PR** | **Min–max PR** | **PR ≥1** |
| --- | --- | --- | --- | --- |
| 0.90 / 0.90 | 1.00 | 0.915 | 0.881–0.948 | 0/200 |
| 0.80 / 0.80 | 1.00 | 0.919 | 0.870–0.980 | 0/200 |
| 0.70 / 0.70 | 1.00 | 0.931 | 0.877–0.989 | 0/200 |
| 1.00 / 1.00 | 0.98 | 0.909 | 0.893–0.931 | 0/200 |
| 1.00 / 1.00 | 0.95 | 0.911 | 0.885–0.938 | 0/200 |
| 0.80 / 0.80 | 0.95 | 0.926 | 0.864–0.980 | 0/200 |
| 0.70 / 0.70 | 0.95 | 0.932 | 0.859–1.008 | 3/200 |
| 0.90 / 1.00 | 1.00 | 0.913 | 0.901–0.924 | 0/200 |
| 0.80 / 1.00 | 1.00 | 0.921 | 0.903–0.943 | 0/200 |
| 0.70 / 1.00 | 1.00 | 0.927 | 0.905–0.945 | 0/200 |
| 0.50 / 1.00 | 1.00 | 0.946 | 0.920–0.975 | 0/200 |

Mean PR and minimum–maximum range summarize the 200 realizations. The range is not a 95% confidence interval, and the count with PR ≥1 is a simulation diagnostic rather than a clinical probability. Sensitivity/specificity uncertainty and sampling uncertainty in the original cohort are not represented by this range.

Statistical methods

Each realization independently generated Bernoulli outcomes for individual records. An observed positive was retained with its specified sensitivity; an observed negative became positive with probability 1 − specificity. We refitted the serum albumin model adjusted for age, sex, diabetes duration, HbA1c, systolic blood pressure, BMI and log UACR, and estimated the marginal standardized PR for a one-SD increase in albumin using the fixed complete-case SD and covariate distribution. NumPy default_rng with seed 20260801 was continued across scenarios in table order.

Interpretation. Scenario mean PRs ranged from 0.909 to 0.946 and were all below one. At sensitivity 0.70 and specificity 0.95, 3 of 200 realizations had PR ≥1; no other scenario did. The table was recalculated using independent per-record false-positive draws and replaces the earlier values. These results describe sensitivity of the albumin model-3 estimate to the specified perturbations; they do not estimate actual ascertainment bias, recover true DR prevalence or test the full M2-versus-M3 comparison.

**Table S14. Sensitivity analysis using the 2021 CKD-EPI creatinine equation**

**A. Models using the alternative eGFR estimate**

This sensitivity analysis retained the 3,978 complete cases, including 958 with documented DR. The primary analysis used hospital-reported eGFR generated with the 2009 CKD-EPI equation. Only the continuous eGFR covariate in M2 and M4 was replaced; M1 and M3 contain no eGFR and are unchanged. All four primary blood markers are reported below.

| **Marker** | **Model** | **PR (95% CI)** | **P** |
| --- | --- | --- | --- |
| Serum albumin | M2 | 0.789 (0.747–0.830) | <0.001 |
| Serum albumin | M4 | 0.919 (0.871–0.966) | 0.002 |
| Total bilirubin | M2 | 0.858 (0.809–0.903) | <0.001 |
| Total bilirubin | M4 | 0.931 (0.880–0.978) | 0.006 |
| Fibrinogen | M2 | 1.110 (1.052–1.165) | <0.001 |
| Fibrinogen | M4 | 0.979 (0.925–1.029) | 0.426 |
| FIB-4 | M2 | 1.091 (1.027–1.151) | 0.003 |
| FIB-4 | M4 | 1.082 (1.025–1.136) | 0.003 |

**Statistical methods**

The 2021 creatinine equation used recorded age in years, sex (0 female, 1 male) and serum creatinine converted from μmol/L to mg/dL by division by 88.4. Intermediate values and the resulting eGFR were not rounded. The equation was implemented as follows, without a race coefficient.

eGFR = 142 × min(SCr/κ, 1)^α × max(SCr/κ, 1)^−1.200 × 0.9938^age × 1.012 (if female). Here κ = 0.7 for women and 0.9 for men; α = −0.241 for women and −0.302 for men. SCr is in mg/dL and eGFR is in mL/min/1.73 m².

M2 adjusted for age, sex, diabetes duration, HbA1c, systolic blood pressure, BMI and the recalculated eGFR; M4 additionally included log UACR. Logistic models and marginal standardization were used as in the primary analysis. Marker contrasts used the original complete-case standard deviations, with natural-log transformation of bilirubin, fibrinogen and FIB-4. Each PR compares predictions after a one-SD increase in the marker with predictions at the observed value, averaged over the same sample.

Confidence intervals were obtained by exponentiating the 2.5th and 97.5th percentiles of 1,000 bootstrap log PRs. Two-sided P values used a normal reference distribution for log(PR) divided by its bootstrap standard error; these exploratory P values are unadjusted. NumPy default_rng with seed 20260801 generated shared participant resamples across models. The calculation used the recorded age, sex and creatinine values; assay calibration and unrounded laboratory-system inputs were unavailable.

**B. Paired model comparisons and threshold membership**

The first 1,000 resamples from the main M1/M3 analysis and the alternative-eGFR M2 analysis used the same seed, generator, sample size and row order. Paired contrasts therefore preserve covariance. Intervals for PR(M3)/PR(M2) use percentiles of the paired log PR difference, followed by exponentiation. The full four-marker comparison is shown; a ratio above one indicates movement toward the null only when interpreted with the underlying PRs.

| **Marker** | **PR(M3)/PR(M2)** | **95% CI** |
| --- | --- | --- |
| Serum albumin | 1.149 | 1.117–1.184 |
| Total bilirubin | 1.067 | 1.041–1.093 |
| Fibrinogen | 0.895 | 0.871–0.917 |
| FIB-4 | 0.993 | 0.971–1.015 |

Attenuation was 100 × [1 − |log PR(adjusted)| / |log PR(M1)|]. Quantitative attenuation reporting remains restricted to albumin and bilirubin. The other marker ratios are reported above without promoting unstable percentage attenuation estimates. Differences below are UACR attenuation minus eGFR attenuation; confidence limits are paired bootstrap percentiles, not causal mediation intervals.

| **Marker** | **eGFR attenuation %** | **UACR attenuation %** | **Difference in percentage points (95% CI)** |
| --- | --- | --- | --- |
| Serum albumin | 35.1 | 73.3 | 38.2 (30.2–48.3) |
| Total bilirubin | 44.5 | 67.9 | 23.4 (15.0–33.2) |

For albumin, the eGFR-adjustment attenuation was 34.3% with the reported eGFR and 35.1% with the 2021 estimate; for bilirubin it was 43.6% and 44.5%, respectively. UACR-adjustment estimates were unchanged by design. The positive paired differences support the continuous-adjustment comparison in this fixed sample.

| **Threshold (mL/min/1.73 m²)** | **Hospital-reported eGFR n** | **2021 eGFR n** | **Moved from below to at/above threshold n** |
| --- | --- | --- | --- |
| ≥60 | 3,111 | 3,212 | 101 |
| ≥90 | 1,817 | 2,088 | 271 |

Threshold counts are within the same 3,978 participants; no participant crossed either threshold in the opposite direction. These are descriptive reclassification counts. Models restricted to the newly defined ≥60 or ≥90 groups and renal-phenotype contrasts were not refitted. Thus these results do not establish robustness of threshold-based analyses to equation choice or compare either equation with measured GFR.

**Equation source**

Inker LA, Eneanya ND, Coresh J, Tighiouart H, Wang D, Sang Y, et al. New Creatinine- and Cystatin C-Based Equations to Estimate GFR without Race. N Engl J Med. 2021;385(19):1737–1749. doi:10.1056/NEJMoa2102953. Implementation and unit conversion were checked against the NIDDK eGFR Equations for Adults documentation (2021 CKD-EPI creatinine equation).
